# Dr. Joseph D. Bryant

**Published:** 1914-05

**Authors:** 


**Dr. Joseph D. Bryant,** Bellevue 1868, of New York, died April 7, aged nearly 69. He was born at East Troy, Wis., March 12, 1845. Following his graduation, he served as interne at Bellevue for two years, then became an assistant to the chair of anatomy and was promoted, step by step till, in 1898, at the merging of the University and Bellevue Hospital Medical Colleges he became full Professor of Surgery. In 1873 he was made a surgeon in the 71st Regiment, N. G. N. Y. In 1882, as Brigadier-General and Surgeon General of the State, he reorganized the medical staff of the National Guard. from which he resigned in 1895. As an author, his most noted achievements are his Operative Surgery and the American System of Surgery, in which he co-operated with Buck. Of his numerous affiliations with professional organizations, we need note only that he was president of the N. Y. State Medical Association in 1898, and of the United Medical Society of the State of N. Y. in 1906. His death was due to diabetes of long standing, in spite of which he carried on a large practice and maintained an interest in professional affairs. Like most men of prominence, he was affable, kindly in nature, and beloved by patients and professional colleagues.

